# Isolation and Evaluation of Indigenous Isolates of *Beauveria bassiana* and Synergistic Control of *Spodoptera frugiperda* with the Parasitoid *Microplitis prodeniae*

**DOI:** 10.3390/insects15110877

**Published:** 2024-11-08

**Authors:** Ling-Wei Zhang, Fang-Fang Lu, Lu Zhu, Chen-Xu Zhou, Xiao-Miao Xu, Nan Zhang, Li-Jun Zhou, Nicolas Desneux, Yao-Hui Wang, Yong-Cheng Dong

**Affiliations:** 1 Key Laboratory of Biology and Sustainable Management of Plant Diseases and Pests of Anhui Higher Education Institutes, School of Plant Protection, Anhui Agricultural University, Hefei 230036, Anhui, China; lwzhang0803@163.com (L.-W.Z.); 19956061728@163.com (F.-F.L.); zhulu1063@163.com (L.Z.); xuxiaomiao98@stu.ahau.edu.cn (X.-M.X.); zhangn0308@163.com (N.Z.); ljzhou0720@163.com (L.-J.Z.); 2 Hexian Agro-Tech Service Center, Ma’anshan 238201, Anhui, China; hxzbzjzzcx@163.com; 3 Université Cote d’Azur, INRAE, UMR ISA, 06000 Nice, France; nicolas.desneux@inrae.fr

**Keywords:** entomopathogenic fungi, biological control, mycopesticide, natural enemies, synergistic effects

## Abstract

Entomopathogenic fungi (EPFs) are capable of infecting a variety of insect pests and are widely used as biopesticides worldwide. This study intended to isolate indigenous EPFs from cadavers of *Protaetia brevitarsis* and investigate their effects on the fall armyworm *Spodoptera frugiperda* (FAW), a globally widespread invasive pest. Morphological and molecular analyses confirmed four native EPF strains all belong to *Beauveria bassiana*. Pathogenicity tests showed they were virulent toward FAW 1st instar larvae. The application of EPFs either by dipping or spraying significantly increased the larval mortalities compared to the control group, with corrected mortalities ranging from 92% to 73% after dipping in a fungal suspension of 10^8^ conidia/mL, and those ranging from 76% to 35% after spraying with a fungal suspension of 10^7^ conidia/mL. Our findings revealed the infectivity of four strains to FAW larvae significantly changed in a dose- and time-dependent manner. In addition, the combination use of the local *B. bassiana* strain and parasitoid *Microplitis prodeniae* resulted in a significantly enhanced *S. frugiperda* 3rd instar larval mortality compared to a single inoculation with one of them, suggesting an apparent synergistic effect for the joint application of these two biological control agents. The mortality inflicted by *B. bassiana* was probably promoted by the release of parasitoids, since the parasitoids’ movements and attacks could strengthen the fungal distribution and infection processes. This study underscores the potential of the combination use of EPFs and parasitoids against *S. frugiperda* early-instar larvae, and provides insights into the consequences of integrating these EPFs into integrated pest management systems.

## Simple Summary

This study confirmed four indigenous isolates of entomopathogenic fungi (EPFs) from the cadavers of *Protaetia brevitarsis*, which are highly virulent toward fall armyworm (FAW) *Spodoptera frugiperda* larvae. Larval mortality caused by these EPFs was affected by the factors of isolate, fungal concentration, application method, and duration. Additionally, the combination use of EPFs and parasitoids could result in a significantly enhanced *S. frugiperda* larval mortality, suggesting the joint application of fungi and parasitoids has a synergistic effect on FAW larval mortality. This knowledge will facilitate the development of biological control against this invasive pest.

## 1. Introduction

The fall armyworm (FAW) *Spodoptera frugiperda* (Smith) (Lepidoptera: Noctuidae) is a highly polyphagous pest that can feed on more than 350 host plants and causes serious damage to several main crops, e.g., maize, rice, sorghum, sugarcane, and cotton [1,2,3]. This pest is an invasive species native to the Americas and has now spread to most territories worldwide because of its massive migratory strength [1,4]. With a wide geographic distribution, the frequent occurrence of this pest poses a considerable threat to crop production [5,6]. For instance, in maize, FAW larvae usually feed inside the whorl leaves and can incur crop yield reductions of up to 70% during the early stages [7]. Therefore, a variety of routine prevention and emergency treatment measures, including agronomic, biological, and chemical approaches, have been adopted to constrain its migration and outbreak around the world [1]. As the predominant and quickest approach, chemical methods have been frequently applied, and various synthetic pesticides have been used. Unfortunately, frequent exposure to high doses of synthetic pesticides has promoted the development of pesticide resistance over the years [8,9,10,11]. In consequence, developing sustainable management measures against FAW is critical to overcoming the problem of serious pesticide resistance and unsatisfactory control [12,13].

Biological control is an important part of integrated pest management (IPM) strategies including the application of pathogens (fungi, bacteria, nematodes, etc.) [14,15,16] and natural enemies (parasitic wasps, predators, etc.) [7,17], and it has been demonstrated as a promising practical supplement to synthetic pesticides for prevention and sustainable control [18,19]. The application of biological control agents plays a significant role in the IPM strategies against *S. frugiperda*. The most commonly used biological control agents are entomopathogenic fungi (EPF), which are ubiquitous and show great potential in controlling insect pests [14,20]. Fungal pathogens produce conidia that can attach to the insect cuticle and penetrate the body with the aid of secreted enzymes that finally kill the insect [21,22,23]. So far, more than 800 fungi species have been identified that are virulent to insects [20,24]. Nevertheless, most mycoinsecticides being commercially developed are species of *Beauveria*, *Metarhizium*, and *Isaria* [24,25].

Many EPF isolates have been applied as alternatives to broad-spectrum chemical pesticides all over the world, although the natural infection and control efficacy can vary due to their ability to survive in different kinds of environments [26,27,28]. For instance, the different isolates of *B*. *bassiana* could exhibit different control efficacies against *S. fruigiperda* [21,29,30,31,32]. The inconsistent performance of EPFs is still a challenge due to their establishment and spread of spores. The commercial isolates may not be well adapted to the local environment while native isolates may be able to naturally regulate the pest population. Under field conditions, the dispersion of microbial biocontrol agents by insects, e.g., pollinators, can increase the spread of EPFs and consequently enhance the control against pests. A dispersal strategy with the help of bumblebees carrying dried non-spore-forming microorganisms has been proven successful [33]. The combined application of *B*. *bassiana* and parasitoids has complimentary impacts on the control of *Grapholita molesta*, as the wasps received the fungal conidia from the treated host egg and assisted the disease transmission [34]. Thus, an entomovectoring system could provide a potential solution to better improve the biological control effects, and the compatibility of biological control agents should be seriously taken into account to optimize the IPM strategies [35].

Considering the dual necessity to achieve good prevalence and high effectiveness, as well as the combination use of natural enemies, studies on native insect-pathogenic fungal strains and isolation have been made toward the exploitation of effective biological control agents. The aim of the present investigation was to identify native entomopathogens, and to evaluate the insecticidal effect of different native isolates with different application methods, and the joint application with parasitoids for the management of FAW under particular conditions. The findings of this study will aid in the development of microbial insecticide-based products.

## 2. Materials and Methods

### 2.1. Insect Source and Rearing

Fall armyworm *Spodoptera frugiperda* was originally collected from a field of maize in Huangshan, Anhui, China, and maintained in a climatic chamber (L:D = 14:10 h, 26 ± 1 °C, RH = 60 ± 5%) at the School of Plant Protection, Anhui Agricultural University. Honey and water were supplied to adult moths. The larvae were reared in plastic boxes (17 cm × 11.3 cm × 7.8 cm) with fresh maize leaves up to the 3rd instar and individually reared in 6-well cell culture plates (12.7 cm × 8.55 cm × 2.3 cm) with artificial diet after the 3rd instar.

Parasitoids *Microplitis prodeniae* Rao and Kurian (Hymenoptera: Braconidae) were originally collected from a field of maize in Suzhou, Anhui, which is one of the locally dominant parasitic wasps. It is a solitary koinobiont endoparasitoid, and thus FAW 2nd–3rd instar larvae were provided for the rearing of the colony in the laboratory.

### 2.2. Isolation and Identification of Entomopathogenic Fungi

Entomopathogenic fungi were isolated from the cadavers of *Protaetia brevitarsis* larvae (Coleoptera: Scarabaeidae), which are saprophagous and intentionally utilized as an ‘insect bioreactor’ in our group for converting agricultural wastes. However, the fungi led to a large number of deaths of *P. brevitarsis* larvae during the mass-rearing processes. The cadavers of *P. brevitarsis* larvae with white muscardine disease were thus collected for the subsequent pure culture and identification as well as further studies in the laboratory.

Morphological observation and molecular methods were used to identify this fungus. Firstly, the fungi were inoculated to potato dextrose agar (PDA) and cultured for one week under 26 ± 1 °C in darkness. Morphological observations were performed to determine the color, shape, growth pattern, elevation, and fungal structures. The photos were captured using a microscope (Nikon Ti S, Tokyo, Japan) by program SMZ1500/DS R12. Secondly, genomic DNA was extracted from fresh fungal mycelium cultured on PDA at 28 °C for a week following the instruction of TransDirect^®^ Plant Tissue PCR Kit (TransGen Biotech, Beijing, China). The internal transcribed spacer (ITS) sequences were amplified using polymerase chain reaction (PCR) with the primers ITS1 (5′-TCCGTAGGTGAACCTGCGG-3′) and ITS4 (5′-TCCTCCGCTTATTGATATGC-3′) following the conditions: initial denaturation at 94 °C for 3 min; with 35 cycles of denaturation at 94 °C for 30 s, annealing at 52 °C for 30 s and extension at 72 °C for 45 s; and final extension at 72 °C for 5 min. PCR products were sent for sequencing (Sangon Biotech, Shanghai, China). Four fungal isolates were cultured and purified from infected cadavers of *P. brevitarsis* larvae, and named BAAU1, BAAU2, BAAU3, and BAAU4. The ITS sequences from different strains were retrieved and compared using the NCBI BLAST search, and a phylogenetic tree containing these four strains and other previously reported *Beauveria* species was constructed using the neighbor-joining method.

### 2.3. Fungal Inoculum Preparation

The mass sporulating culture of each isolate was maintained on PDA for two weeks at 26 ± 1 °C in the dark, and then stock suspension was prepared by adding distilled water with 0.01% Tween-80. A sterilized cotton swab was used to gently wipe the surface to dislodge the conidia. The mixture was then filtered to remove hyphae and residues. The conidial concentration was determined under the microscope with a hemocytometer. Four concentrations of 1 × 10^5^, 1 × 10^6^, 1 × 10^7^, and 1 × 10^8^ conidia mL^−1^ were prepared by dissolving the stock suspension with sterilized distilled water with 0.01% Tween-80 and used for the following bioassay.

### 2.4. Pathogenicity Test

Two common application methods, i.e., dipping and spraying, were adopted to test for pathogenicity. One-day-old FAW 1st instar larvae were randomly selected and subjected to the bioassay. The larvae were dipped for 3–5 s or uniformly sprayed with different fungal concentrations using a spray bottle. Four fungal concentrations (1 × 10^5^, 1 × 10^6^, 1 × 10^7^, and 1 × 10^8^ conidia mL^−1^) were used for dipping while three fungal concentrations (1 × 10^5^, 1 × 10^6^, and 1 × 10^7^ conidia mL^−1^) were applied for spraying since it was hard to obtain enough solution with 1 × 10^8^ mL^−1^ conidia concentration. Sterilized distilled water containing 0.01% Tween-80 was used as a control. After dipping or spraying, the larvae were placed on sterilized filter paper to eliminate the excess water and then were transferred into a plastic box (10 cm × 6 cm × 4 cm, with mesh on the lid) with fresh maize leaves that were supplied daily. The experiment had four replicates for each concentration and each replicate contained 20 larvae in a box. Larvae were placed under L:D = 14:10 h, 26 ± 1 °C, RH = 60 ± 5% condition, and the mortality was daily checked and recorded for one week.

### 2.5. Combination Use of Entomopathogenic Fungi and Parasitic Wasp

The combination use of BAAU2 with high virulence and *M. prodeniae* was applied to test the effects of these two biological control measures on the control of FAW in a laboratory cup experiment (for dipping) and greenhouse pot experiment (for spraying). There were four treatments under each condition including (1) only use of BAAU2; (2) only release of *M. prodeniae*; (3) first use of BAAU2 and then release of *M. prodeniae* on the second day; (4) first release of *M. prodeniae* and then use of BAAU2 on the second day. Twenty 3rd instar larvae of FAW were randomly selected for each treatment because the 3rd instar stage is the most suitable for *M. prodeniae*. Each treatment was replicated four times. Under laboratory conditions, the FAW larvae were dipped into fungal suspension at concentration of 1 × 10^8^ conidia mL^−1^ and then placed in the plastic cup (Diameter: 8 cm; Height: 15 cm) with fresh maize leaves, which was covered by the mesh; while the fungal suspension at concentration of 1 × 10^7^ conidia mL^−1^ was sprayed to the FAW larvae on the potted maize plant covered by a microcosm consisting of a transparent polyethylene cylinder (diameter: 20 cm; height: 60 cm) with vented openings in the middle and top covered by fine mesh. One pair of *M. prodeniae* male and female adults were released into the cup or microcosm accordingly. The FAW larval status and the number of emerged cocoons were daily checked and recorded for one week.

### 2.6. Statistical Analysis

Cumulative mortality (CM) and corrected cumulative mortality (CCM) were calculated according to the following equation:

$$\mathrm{CM}=\frac{\mathrm{The}\;\mathrm{cumulative}\;\mathrm{number}\;\mathrm{of}\;\mathrm{dead}\;\mathrm{larvae}}{\mathrm{The}\;\mathrm{total}\;\mathrm{number}\;\mathrm{of}\;\mathrm{treated}\;\mathrm{larvae}}\times 100\%\;\mathrm{CCM}=\frac{\mathrm{CM}(\mathrm{treated}\;\mathrm{group})-\mathrm{CM}(\mathrm{control}\;\mathrm{group})}{1-\mathrm{CM}(\mathrm{control}\;\mathrm{group})}\times 100\%$$

Two-way repeated measures ANOVA was used to test the effects of the factors of strain, and concentration on the survival of FAW larvae, as well as the factors of parasitoid and EPFs on the survival of FAW larvae, followed by Tukey–Kramer HSD post hoc test for multiple comparisons when necessary. Data sets were tested for normality and homogeneity of variance using Kolmogorov–Smirnov D test and Cochran’s test, respectively, and the cumulative mortality and corrected cumulative mortality were arcsin square-root-transformed. Survival curves of FAW larvae were subjected to a Kaplan–Meier analysis and log-rank test. All these data were analyzed using SPSS 16.0 (SPSS Inc., Chicago, IL, USA).

## 3. Results

### 3.1. Identification of Entomopathogenic Fungi

**Figure 1:**
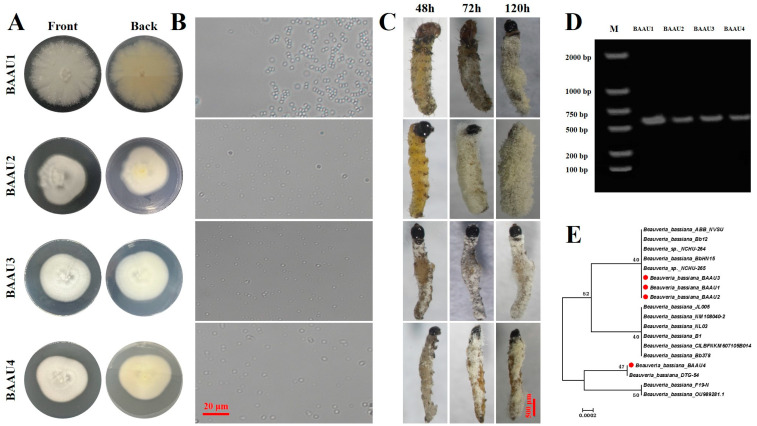
Morphological characteristics of BAAU1-4 colonies: (**A**) macroscopic observation on the top and bottom colonies on PDA medium; (**B**) microscopic observation of conidiophores; (**C**) the mycosis caused on *Spodoptera frugiperda* larvae at each indicated time point; (**D**) electrophoresis of PCR amplified ITS region from four native entomopathogenic fungal isolates; and (**E**) phylogenetic tree of the isolated strains and other related *Beauveria bassiana* strains based on ITS sequence (Neighbor-Joining method). Four native *Beauveria bassiana* strains were indicated with red dots.

Four fungal isolates BAAU1, BAAU2, BAAU3, and BAAU4 were obtained from *P. brevitarsis* larvae cadavers after morphological and molecular identification. Four isolates had the typical morphological traits of the *Beauveria* genus in macroscopic and microscopic observations (Figure 1A,B), displaying a white-to-yellowish color on the mycelium with short and globose conidiophores (Table 1 and Figure 1B). BAAU1 was round while the other three isolates BAAU2-4 were oval in shape. All four strains shared a high similarity to *Beauveria*.

The ITS sequences of four isolates were cloned and sequenced (NCBI GenBank accession number: BAAU1: PQ482683.1; BAAU2: PQ482684.1; BAAU3: PQ482685.1; BAAU4: PQ482686.1) (Figure 1D), and the phylogenetic tree was thus constructed comprising of 4 novel native isolates and other 14 sequences of *Beauveria* strains, showing a very close relationship to the *Beauveria* genus (Figure 1E). By combining the morphological and molecular analyses, we confirmed the four isolated fungi are isolates of *Beauveria bassiana*.

**Table 1:** The macroscopic colony characteristics of four isolates of *Beauveria bassiana*.

Isolate	Morphological Characteristics				
	Color *	Shape	Growth Pattern	Elevation	Texture
BAAU1	White/Yellowish	Round	Disperse	Raised	Smooth
BAAU2	White/Yellowish-brown	Oval	Dense and disperse	Raised	Smooth
BAAU3	White/Brown	Oval	Dense and disperse	Raised	Smooth
BAAU4	White/Yellowish-brown	Oval	Dense and disperse	Raised	Smooth

* Colony color on the top/bottom side.

### 3.2. Pathogenicity Test

**Figure 2:**
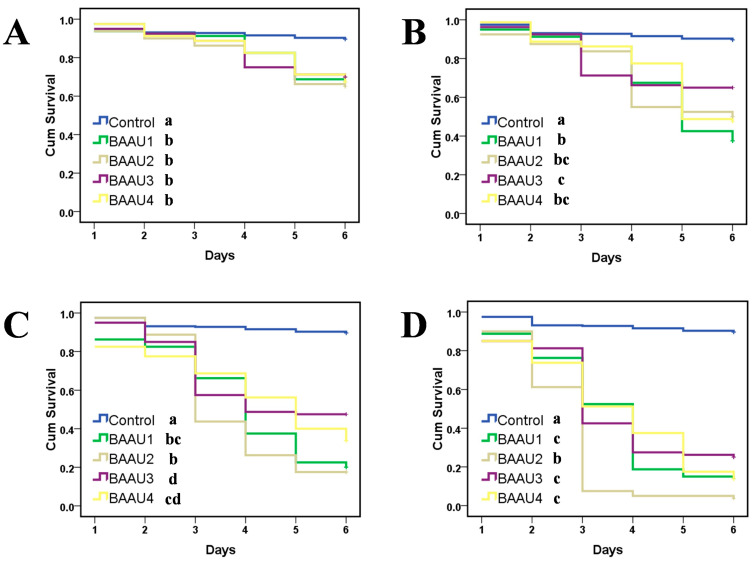
Survival curves of *Spodoptera frugiperda* larvae caused by the four *Beauveria bassiana* strains BAAU1, BAAU2, BAAU3, and BAAU4 at four fungal concentrations: (**A**) 1 × 10^5^, (**B**) 1 × 10^6^, (**C**) 1 × 10^7^ and (**D**) 1 × 10^8^ conidia mL^−1^ by dipping method. The groups of larvae treated with sterilized distilled water with 0.01% Tween-80 serve as control. Curves with the same letters are not significantly different (Log Rank (Mantel–Cox) test, *p* > 0.05).

**Figure 3:**
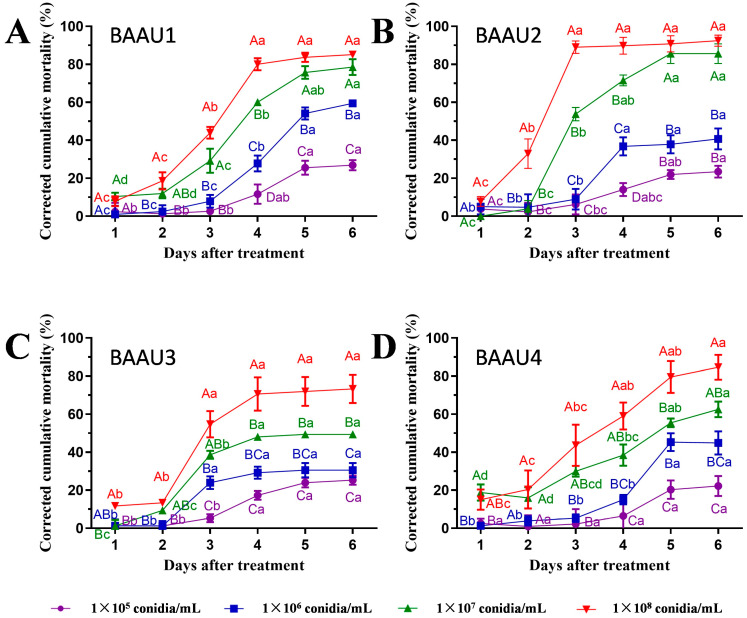
Corrected cumulative mortalities (Mean ± SE) of *Spodoptera frugiperda* larvae caused by the four *Beauveria bassiana* strains: (**A**) BAAU1, (**B**) BAAU2, (**C**) BAAU3, (**D**) BAAU4 at different concentrations by dipping method. Mean values for the isolate with a certain concentration across different days bearing the same lower-case letters and with different concentrations on a certain day bearing the same upper-case letters are not significantly different (Tukey test, *p* > 0.05).

**Figure 4:**
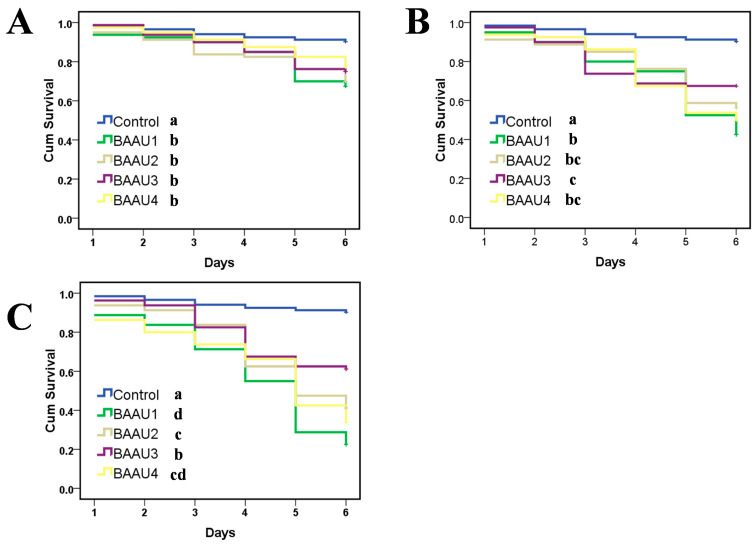
Survival curves of *Spodoptera frugiperda* larvae caused by the four *Beauveria bassiana* strains BAAU1, BAAU2, BAAU3, and BAAU4 at three fungal concentrations: (**A**) 1 × 10^5^, (**B**) 1 × 10^6^, and (**C**) 1 × 10^7^ conidia mL^−1^ by spraying method. The groups of larvae treated with sterilized distilled water with 0.01% Tween-80 serve as control. Curves with the same letters are not significantly different (Log Rank (Mantel–Cox) test, *p* > 0.05).

**Figure 5:**
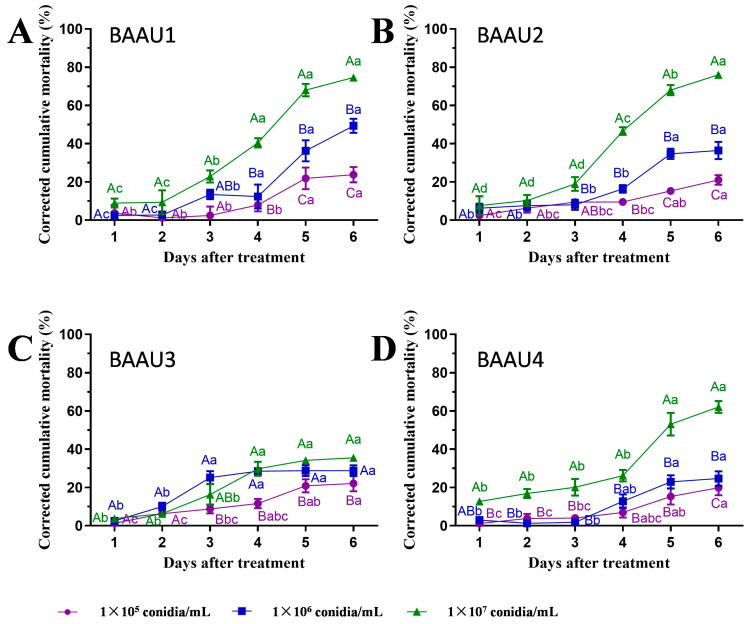
Corrected cumulative mortalities (Mean ± SE) of *Spodoptera frugiperda* larvae caused by the four *Beauveria bassiana* strains: (**A**) BAAU1, (**B**) BAAU2, (**C**) BAAU3, (**D**) BAAU4 at different concentrations by spraying method. Mean values for the isolate with a certain concentration across different days bearing the same lower-case letters and with different concentrations on a certain day bearing the same upper-case letters are not significantly different (Tukey test, *p* > 0.05).

The application of these four isolates rapidly killed the FAW larvae and led to a quick mycosis on the larval body within two days (Figure 1C), exhibiting great potential for controlling FAW. The insect bodies of FAW were full of fungal mycelium at three days post-inoculation. The statistical analyses of pathogenicity tests showed that the factors strains and concentrations significantly affected the mortality (both cumulative and corrected cumulative mortality) of FAW larvae in either dipping or spraying experiment (*p* < 0.001), and there was significant interaction across these two factors (*p* < 0.05) (Table 2, Table 3, Table 4 and Table 5). The results revealed that the virulence of the four native strains was significantly different. Their efficacies were significantly increased along with the increasing concentration and days (Table 2, Table 3, Table 4 and Table 5, Figure 2, Figure 3, Figure 4 and Figure 5). That is, the infectivity of the four strains to FAW larvae significantly changed in a dose- and time-dependent manner, with the highest mortality occurring at the highest concentration over the days (Figure 2, Figure 3, Figure 4 and Figure 5). The application methods of isolates (either by dipping or spraying) resulted in high mortality of FAW larvae (Figure 2, Figure 3, Figure 4 and Figure 5).

#### 3.2.1. Dipping Treatment

Compared with the control group (survival > 90% in a week), dipping in the fungal suspension resulted in a rapid death of FAW larvae and four isolates showed significantly different virulence (Log Rank (Mantel–Cox) test: for 1 × 10^4^ conidial/mL: chi-square = 44.793, *df* = 4, *p* < 0.001, Figure 2A; for 1 × 10^5^ conidial/mL: chi-square = 123.222, *df* = 4, *p* < 0.001, Figure 2B; for 1 × 10^6^ conidial/mL: chi-square = 257.264, *df* = 4, *p* < 0.001, Figure 2C; for 1 × 10^8^ conidial/mL: chi-square = 408.766, *df* = 4, *p* < 0.001, Figure 2D). Larval cumulative mortalities and corrected cumulative mortalities were increased along the duration (Figure 2) and positively correlated with the fungal suspension concentration in all dipping treatments (Table 2, Figure 3). The corrected cumulative mortalities of four strains at 6 days after dipping in a fungal suspension of 10^8^ conidia/mL had no significant difference ranging from 73% to 92% (*F*_3,12_ = 2.296, *p* = 0.130). However, compared with the other three isolates, BAAU2 showed the quickest control effects as it needed the shortest time to reach the highest larval mortality (Table 3, Figure 2).

**Table 2:** Statistics from the two-way repeated measures ANOVA used to analyze the cumulative mortality and corrected cumulative mortality of *Spodoptera frugiperda* larvae in the dipping trial.

Source of Variation	Cumulative Mortality			Corrected Cumulative Mortality		
	*df*	*F*	*Sig*.	*df*	*F*	*Sig*.
*Strains*	3	13.091	<0.001	3	10.101	<0.001
*Concentrations*	3	129.113	<0.001	3	129.786	<0.001
*Strains × Concentrations*	9	2.693	0.013	9	2.999	0.006
*Error*	48			48		

**Table 3:** Cumulative mortality at day 6, mean and median values of survival time of *Spodoptera frugiperda* larvae after dipping in the suspension of four *Beauveria bassiana* strains at different concentrations. The groups of larvae treated with sterilized distilled water with 0.01% Tween-80 serve as control.

Isolates	Concentration (Conidia mL^−1^)	Mortality * ± SE	Survival Time			
			Mean ± SE	95% Confidence Interval	Median ± SE	95% Confidence Interval
Control	/	0.103 ± 0.017	5.653 ± 0.065	5.526–5.578	-	-
BAAU1	10^5^	0.325 ± 0.014	5.288 ± 0.155	4.983–5.592	-	-
	10^6^	0.625 ± 0.014	4.825 ± 0.156	5.132–5.000	5.000 ± 0.221	4.567–5.433
	10^7^	0.800 ± 0.041	3.950 ± 0.182	3.594–4.306	4.000 ± 0.188	3.631–4.369
	10^8^	0.863 ± 0.013	3.512 ± 0.165	3.189–3.836	4.000 ± 0.152	3.703–4.297
BAAU2	10^5^	0.350 ± 0.035	5.112 ± 0.171	4.778–5.447	-	-
	10^6^	0.500 ± 0.035	4.712 ± 0.018	4.360–5.065	-	-
	10^7^	0.875 ± 0.048	3.738 ± 0.148	3.448–4.027	3.000 ± 0.123	2.758–3.242
	10^8^	0.938 ± 0.024	2.688 ± 0.116	2.460–2.915	3.000 ± 0.055	2.893–3.107
BAAU3	10^5^	0.300 ± 0.020	5.225 ± 0.160	4.910–5.540	-	-
	10^6^	0.350 ± 0.029	4.912 ± 0.176	4.567–5.258		
	10^7^	0.525 ± 0.014	4.337 ± 0.191	3.963–4.712	-	-
	10^8^	0.750 ± 0.071	3.625 ± 0.189	3.255–3.995	3.000 ± 0.143	2.720–3.280
BAAU4	10^5^	0.325 ± 0.032	5.312 ± 0.150	5.018–5.607	-	-
	10^6^	0.525 ± 0.032	5.000 ± 0.151	4.705–5.295	-	-
	10^7^	0.675 ± 0.032	4.250 ± 0.213	3.832–4.668	5.000 ± 0.337	4.339–5.661
	10^8^	0.863 ± 0.055	3.650 ± 0.189	3.280–4.020	4.000 ± 0.299	3.415–4.585

* indicates the cumulative mortality on day 6.

#### 3.2.2. Spraying Treatment

The spraying of four isolates led to a significantly increased mortality in all treated groups (Log Rank (Mantel–Cox) test: for 1 × 10^4^ conidial/mL: chi-square = 35.813, *df* = 4, *p* < 0.001, Figure 4A; for 1 × 10^5^ conidial/mL: chi-square = 112.407, *df* = 4, *p* < 0.001, Figure 4B; for 1 × 10^6^ conidial/mL: chi-square = 211.630, *df* = 4, *p* < 0.001, Figure 4C). Similarly, larval cumulative mortalities and corrected cumulative mortalities were significantly different across fungal suspension concentrations (Figure 5) and durations (Figure 4) in the spraying groups. After spraying an EPF suspension of 10^7^ conidia/mL, FAW larval corrected cumulative mortalities at 6 days ranged from 35% to 76% (*F*_3,12_ = 90.008, *p* < 0.0001).

Compared with the dipping groups, the spraying groups exhibited slow and reduced control effects against FAW larvae (Table 3 and Table 5; Figure 2, Figure 3, Figure 4 and Figure 5). Considering the results from two application methods together, BAAU1 and BAAU2 were considered more virulent than the other two strains, and thus BAAU2 was selected for the following combined pest control experiment.

**Table 4:** Statistics from the two-way repeated measures ANOVA used to analyze the cumulative mortality and corrected cumulative mortality of *Spodoptera frugiperda* larvae in the spraying trial.

Source of Variation	Cumulative Mortality			Corrected Cumulative Mortality		
	*df*	*F*	*Sig*.	*df*	*F*	*Sig*.
*Strains*	3	20.646	<0.001	3	7.186	<0.001
*Concentrations*	2	161.268	<0.001	2	162.864	<0.001
*Strains × Concentrations*	6	8.976	0.001	6	9.302	<0.001
*Error*	36			36		

**Table 5:** Cumulative mortality at day 6, mean and median values of survival time of *Spodoptera frugiperda* larvae after being treated with four *Beauveria bassiana* strains at different concentrations. The groups of larvae treated with sterilized distilled water with 0.01% Tween-80 serve as control.

Isolates	Concentration (Conidia mL^−1^)	Mortality * ± SE	Survival Time			
			Mean ± SE	95% Confidence Interval	Median ± SE	95% Confidence Interval
Control	/	0.097 ± 0.010	5.728 ± 0.054	5.621–5.835	-	-
BAAU1	10^5^	0.325 ± 0.025	5.312 ± 0.156	5.007–5.618	-	-
	10^6^	0.575 ± 0.014	4.925 ± 0.168	4.595–5.255	6.000 ± 0.340	5.333–6.667
	10^7^	0.775 ± 0.014	4.275 ± 0.184	3.914–4.636	5.000 ± 0.193	4.622–5.378
BAAU2	10^5^	0.300 ± 0.020	5.288 ± 0.167	4.961–5.641	-	-
	10^6^	0.438 ± 0.031	5.000 ± 0.178	4.652–5.348	-	-
	10^7^	0.788 ± 0.013	4.788 ± 0.166	4.461–5.114	5.000 ± 0.525	3.970–6.030
BAAU3	10^5^	0.263 ± 0.024	5.438 ± 0.137	5.169–5.706	-	-
	10^6^	0.325 ± 0.014	4.975 ± 0.175	4.632–5.318	-	-
	10^7^	0.388 ± 0.013	5.025 ± 0.163	4.706–5.344	-	-
BAAU4	10^5^	0.288 ± 0.043	5.538 ± 0.134	5.276–5.799	-	-
	10^6^	0.500 ± 0.035	4.938 ± 0.163	4.617–5.258	6.000	-
	10^7^	0.663 ± 0.031	4.488 ± 0.203	4.089–4.886	5.000 ± 0.233	4.544–5.456

* indicates the cumulative mortality on day 6.

### 3.3. Combination Effects of Using BAAU2 and Microplitis prodeniae

**Figure 6:**
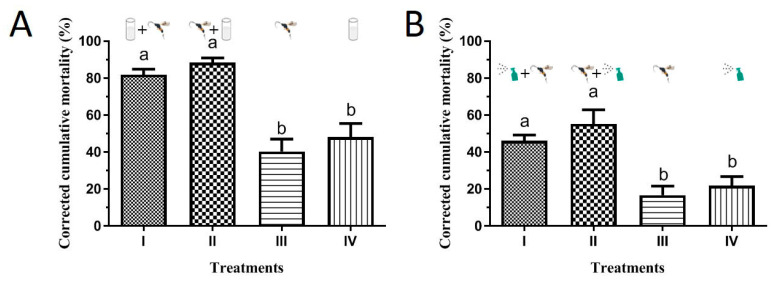
Corrected mortalities (Mean ± SE) of *Spodoptera frugiperda* larvae caused by the use of *Microplitis prodeniae* and/or *Beauveria bassiana* isolate BAAU2 strain in (**A**) laboratory cup and (**B**) greenhouse pot experiment. BAAU2 strain with the concentration of 10^8^ conidia/mL was used for dipping in the cup experiment and with the concentration of 10^7^ conidia/mL was used for spraying pot experiment. Treatments are as follows: (I) *M. prodeniae* was released after the inoculation of BAAU2 strain; (II) *M. prodeniae* was first released before the inoculation of BAAU2 strain; (III) Only release of *M. prodeniae*; (IV) Only inoculation of BAAU2 strain. Bars bearing the same letters are not significantly different (ANOVA, *p* > 0.05).

**Figure 7:**
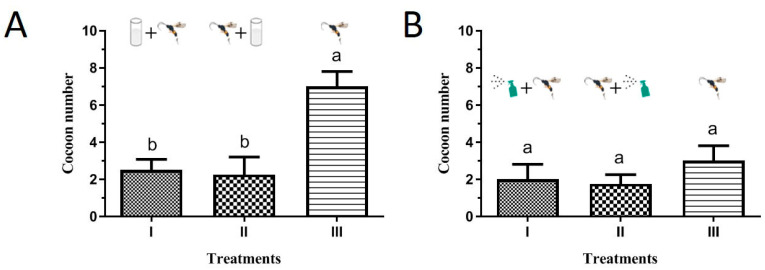
Cocoon number (Mean ± SE) of *Microplitis prodeniae* from different treatments in (**A**) laboratory cup and (**B**) greenhouse pot experiment. BAAU2 strain with the concentration of 10^8^ conidia/mL was used for dipping in the cup experiment and with the concentration of 10^7^ conidia/mL was used for spraying pot experiment. Treatments are as follows: (I) *M. prodeniae* was released after the inoculation of the BAAU2 strain; (II) *M. prodeniae* was first released before the inoculation of the BAAU2 strain; (III) Only release of *M. prodeniae*. Bars bearing the same letters are not significantly different (ANOVA, *p* > 0.05).

The results showed the single use of BAAU2 or *M. prodeniae* resulted in approximate 20~40% death of FAW 3rd instar larvae in the laboratory cup and greenhouse pot experiments, while the combination use of BAAU2 and *M. prodeniae* resulted in a higher rate of FAW larvae death (ranging from 40% to 90%) (Figure 6). In other words, the combination use of BAAU2 and *M. prodeniae* significantly increased the mortality of FAW larvae than either single use of entomopathogenic fungi or parasitoid (laboratory cup experiment: *F*_3,13_ = 19.957, *p* < 0.0001, Figure 6A; greenhouse pot experiment: *F*_3,13_ = 32.535, *p* < 0.0001, Figure 6B). The factors (i.e., *parasitoids* and *EPFs*) significantly affected the survival of FAW 3rd instar larvae in the laboratory cup and greenhouse pot experiments (Laboratory cup: *Parasitoids*: *F*_1,16_ = 28.673, *p* < 0.001, *EPFs*: *F*_1,16_ = 346.327, *p* < 0.001; Greenhouse pot: *Parasitoids*: *F*_1,16_ = 27.673, *p* < 0.001, *EPFs*: *F*_1,16_ = 242.908, *p* < 0.001), and there was a significant interaction between them in both experiments (Laboratory cup: *F*_1,16_ = 14.248, *p* = 0.002; Greenhouse pot: *F*_1,16_ = 17.766, *p* = 0.001), suggesting there was an apparent synergistic effect. The control efficacies in the combination use of two control measures were more than two-fold in the single use of *M. prodeniae* or BAAU2 strain (Figure 6A,B). The offspring number of parasitoids was compared to see the effects of BAAU2 on the development of parasitic wasps. The emerged cocoon numbers of *M. prodeniae* were not different among the four treatments in the pot experiment (*F*_2,9_ = 3.316, *p* = 0.083) (Figure 7B), while it was significantly reduced in the combination use groups in the cup experiment (*F*_2,9_ = 44.739, *p* < 0.0001) (Figure 7A).

## 4. Discussion

The present study aimed to molecularly and morphologically identify four indigenous isolates of entomopathogenic fungi that could be exploited as potential biological control agents for insect pests. They were confirmed as *Beauveria bassiana* from morphological traits and ITS sequences. The pathogenicity tests showed all four native strains were highly virulent to FAW larvae, especially for BAAU1 and BAAU2. Larval mortality rates increased over the time after treatment and positively correlated to the conidia concentration. Both application methods (i.e., dipping and spraying) led to significant control effects against FAW larvae. Moreover, the simultaneous application of *B. bassiana* and *M. prodeniae* resulted in a significantly increased suppression of FAW larvae over that caused by using *B. bassiana* or *M. prodeniae* alone, showing a synergistic interaction between these two biological control methods.

Entomopathogenic fungi live in the soil and serve as an important contributor to affect insect population dynamics [16,21,24,26]. They are prevalent and cause epizootics when the conditions are suitable. During the artificial rearing processes of saprophagous beetle *P. brevitarsis* larvae, we found that the local unknown entomopathogenic fungus outbreak caused the large-scale death of *P. brevitarsis* larvae and pupae. As shown in the present study, these native EPFs were isolated and confirmed as *B. bassiana*, which are well-known pathogens of scarabs as they require the host to grow and sporulate in soil. They could lead to *P. brevitarsis* epidemics during the mass-rearing processes. The pathogenicity results showed these four local EPF isolates were effective in controlling FAW and caused significant larval mortalities, implying the potential for these four native novel EPFs, despite the virulent levels differed depending on the isolate, infection concentration, application method, and infection duration. Indeed, multiple factors including but not limited to isolate, host age, route of infection, and quantity of inoculum have impacts on the virulence of *B. bassiana* [36]. Among them, BAAU1 and BAAU2 were found to be more effective isolates than the other two and could result in >90% larval mortalities in six days, which showed great potential for developing as mycopesticide compared with previous studies [21,31]. However, the controlling effects of their mixtures deserve to be studied since it was proven that the application of *Beauveria* mixtures containing low-virulent and high-virulent isolates increased the overall mortality of diamondback moth than the use of a single isolate, probably due to the different individual requirements of isolates [37].

EPFs from the *Beauveria* genus have received much attention and have been widely applied as commercial bio-pesticides for insect pest control [21,37,38,39,40]. These EPFs have a broad host spectrum including some tested Lepidoptera and Coleoptera larvae and can adapt to the different laboratory and field conditions, as reported in *S. fruigiperda* [21,29,31,32], *Helicoverpa armigera* Hübner (Lepidoptera: Noctuidae) [41], *S. exigua* Hübner (Lepidoptera: Noctuidae) [42], *Monochamus alternatus* Hope (Coleoptera: Cerambycidae) [43], and *Hypothenemus hampei* Ferrari (Coleoptera: Scolytidae) [44]. Our results indicated that the infectivity of four strains to FAW larvae significantly changed in a dose- and time-dependent manner, with the highest mortality occurring at the highest concentration over the days. Larval mortality was positively correlated with conidia concentrations and developmental durations, largely because the fungal infection processes were affected by the number of conidia received on the insect body surface as well as appressoria development and penetration of insect cuticle [21,45,46]. On the other side, the germination (e.g., germination rate, germination time) of EPFs might also affect the control effects because moisture and temperature, e.g., the soaking duration of the fungal suspension, have impacts on the germination synchronization of spores [47]. The delivery manner can significantly change the number of conidia on the insect body surface and thus alter the fate of FAW larvae [36].

Biological control is an extremely supportive approach for IPM, and the compatibility of bio-control measures is an important issue, for example, if entomopathogens and natural insect enemies are simultaneously or jointly employed [18,48,49]. It is worth noting that the control effectiveness of EPFs was significantly enhanced in the presence of parasitic wasps *M. prodeniae*, suggesting a positive correlation between these two biological control agents [48]. The reasons for joint application of EPFs and parasitoids contributing to significantly enhanced control efficacies may be attributed to the following aspects: (1) the increased spread of EPFs by acquisition and host-oriented transmission of parasitic wasps; (2) the enhanced infection success of EPFS via oviposition or host feeding wounds caused by wasps; and (3) the disrupted immune responses of FAW larvae resulting from polydnaviruses (PDV, obligatory symbionts with parasitic wasps) [50,51,52]. Pre- or post-inoculation of parasitoids may cause the cuticle wound that facilitates the infection processes by EPFs and thus significantly increases the larval mortalities (see Figure 4). The wounds from parasitoid oviposition sting or host feeding substantially increase the natural infection of entomopathogenic fungus. On the other side, the symbiotic PDV from parasitic wasps manipulates caterpillar immunity, but allows them to grow faster to improve host suitability for the parasitoid [53]. The PDV virions were injected into the caterpillar host to ensure survival and development of wasp larvae [51]. Consequently, some of them suppress host immune responses [52], disrupt host cellular encapsulation [54], and/or induce apoptosis of host hemocytes [55], which can render them vulnerable to other pathogens. In addition, the movement of parasitoids, especially host-seeking and orientation behavior, enhanced the contact and transmission of the spores of EPFs toward target insect pests. Actually, the poor establishment and limited spread of EPFs are the main challenges restraining biological control efficiency [33]. Therefore, inundative release of parasitic wasps carrying EPF spores facilitates the EPFs’ spore spread precisely and oriented toward the host due to their host-seeking and piercing capacity (sting or biting), and this kind of entomovectoring dispersal strategy may promote pest control efficacy as was evidenced in the other cases [48,52]. Entomovectoring system has now been attempted in pollinators [33], predators [56], parasitoids [34,48], and even in entomopathogenic nematodes [57]. However, the harmful effects of EPFs on beneficial insect vectors should be taken into consideration.

The compatibility of the multiuse of biological agents (e.g., EPF-based pesticides with parasitoids) is the key premeditated component for enhanced IPM of target pest [35]. The relationship between EPFs and parasitic wasps is one crucial aspect that should be taken into consideration as it varied and was reported independently, synergistically, or antagonistically in some cases [58,59,60]. The parasitoid *Sclerodermus guani* carrying high concentrations of *B. bassiana* may incur changes in pre-reproductive time, fertility, offspring survival, and development, causing interspecific competition even though it is able to coexist and communicate on the same host [43]. The fecundity and survival of egg parasitoid *Trichogramma dendrolimi* were negatively correlated with the concentrations of *B. bassiana* [34]. Not only physiological changes, but the application of *B. bassiana* could alter the choice behavior and development of aphid parasitoid *Aphidius colemani* [61]. Chepkemoi et al. (2024) reported that the *B. bassiana* isolate ICIPE 621 negatively affected the survival rather than the emerged offspring and sex ratio of the FAW egg parasitoid *Telenomus remus* [62]. However, the extended host developmental duration caused by parasitism increased their exposure time to pathogens, leading to a higher mortality of pests but also rendering high risk for immature parasitoids [59]. On the other hand, the biosafety assessment should be performed prior to the simultaneous use of EPFs and parasitoids as biological characteristics and pathogenicity of EPFs vary toward parasitic wasps [58,59]. The screening of strains safe from natural enemies will be necessary for the joint application and dry conidia should be recommended [33]. The combination use of BAAU2 and *M. prodeniae* did not affect the parasitoids’ offspring number since no significant decrease in cocoon number was observed in the pot experiment (Figure 7B), implying an ideal compatibility for population establishment in the open field. The likely cause is that *M. prodeniae* can recognize the EPF-infected host and consequently lay eggs in a healthy host. In the laboratory cup experiment, the higher offspring cocoon number was observed in the group of the single use of parasitoids, probably being attributed to the enhanced parasitic capacity resulting from the space limitation. Even though a low virulence of EPFs to parasitoids was observed in some cases [48,58,59], pest mortality in the combination use of both EPFs and parasitoids was far more than the use of EPFs or parasitoids alone, suggesting this application mode is promising for the future control of FAW and other pests. However, the combined use of EPFs and parasitoids was only tested under laboratory and semi-field cage conditions in the present study, and the practical effects will need to be proved in the field.

## 5. Conclusions

In summary, the present study isolated and identified four strains of indigenous entomopathogenic fungi belonging to *B. bassiana*. The pathogenicity test indicated that all four local isolates had high natural infectivity and led to great mortality in the FAW larvae, especially for BAAU1 and BAAU2, implying that they could be used as effective biological control agents to complement the current IPM against FAW. The combination use of local *B. bassiana* strains and *M. prodeniae* created a significantly higher pathogenic effect against FAW, which might reduce the reliance on chemical pesticides and better improve the existing IPM packages.

## Data Availability

All the data that support the findings of this study are available in the manuscript.
